# Work-family life courses and markers of stress and inflammation in mid-life: evidence from the National Child Development Study

**DOI:** 10.1093/ije/dyv205

**Published:** 2015-10-14

**Authors:** Rebecca E Lacey, Amanda Sacker, Meena Kumari, Diana Worts, Peggy McDonough, Cara Booker, Anne McMunn

**Affiliations:** 1 Department of Epidemiology & Public Health, University College London, London, UK; 2 Institute for Social and Economic Research, University of Essex, Colchester, UK and; 3 Dalla Lana School of Public Health, University of Toronto, Toronto, ON, Canada

**Keywords:** National Child Development Study, sequence analysis, inflammation, cortisol, life course, work, partnerships, parenthood

## Abstract

**Background:** This study investigated associations between work-family life courses and biomarkers of inflammation and stress in mid-life among British men and women. Gender differences in these associations were also explored.

**Methods:** A novel statistical method—multi-channel sequence analysis—defined work-family life courses between the ages of 16 and 42 years, combining annual information on work, partnership and parenthood. Associations between work-family life courses and inflammation [C-reactive protein (CRP), fibrinogen and von Willebrand factor] and cortisol at age 44/45 years were tested using multivariate linear regression using multiply-imputed data on almost 6500 participants from the National Child Development Study 1958 British birth cohort.

**Results:** Compared with those who combined strong ties to paid work with later transitions to stable family lives (‘Work, later family’ group), ‘Teen parents’ had higher CRP [40.6% higher, 95% confidence interval (CI): 5.6, 87.0] and fibrinogen (7.8% higher, 95% CI: 2.3, 13.5) levels, and homemakers (‘No paid work, early family’) had raised fibrinogen levels (4.7% higher, 95% CI: 0.7, 9.0), independent of childhood health and socioeconomic position, adult socioeconomic position, health behaviours and body mass index (BMI). Those who combined later transitions to stable family ties with a career break for childrearing had higher post-waking cortisol than the ‘Work, later family’ group; however, no associations were seen for other work-family types, therefore suggesting a null finding with cortisol. No statistically significant gender interactions in associations between work-family types and inflammatory or cortisol outcomes were found.

**Conclusions:** Work-family life courses characterised by early parenthood or weak work ties were associated with a raised risk profile in relation to chronic inflammation.

Key Messages

- Multi-channel sequence analysis was used to derive work-family life courses for men and women in the 1958 British birth cohort.
- Women in this cohort had more diverse work-family life courses than men, typically involving weaker ties to paid work.
- Work-family life courses characterised by earlier transitions to parenthood in combination with weak long-term ties to paid work were associated with increased inflammation in mid-life.
- This suggests that the transition to adulthood is a key life course period from which work and family trajectories might have long-term implications for health.

## Background

Participation in paid work and living in a stable partnership have both been consistently linked with better health outcomes and a lower risk of mortality.^1–8^ However, a widely held contention among life course researchers is that long-term experiences in work and family life are interdependent.^9^^,^^10^ Indeed, family responsibilities have traditionally signified reduced access to paid employment for women,^11–13^ suggesting that the work-family combinations may differently influence the health of women and men. Yet few studies have assessed the health effects of these combined histories; fewer still have incorporated men. Longitudinal research suggests that women who combined paid work and family over the long term end up healthier than those who do not,^5^^,^^14–16^ although this finding is not universal.^17^ For example, a study of British women who are now of retirement age showed that those who combined relatively strong labour market ties with stable marriage were healthier in their mid-50s than those who spent long periods of time out of the labour market looking after the home and family, independent of social position or health earlier in adulthood.^3^ It is hypothesised that the combination of weak ties to paid work, unstable partnerships and early entry to parenthood is associated with poorer health profiles of both men and women.

Another shortcoming of existing research is its reliance on subjective measures of health. Hypothalamic-pituitary-adrenal (HPA) axis functioning and chronic low-grade inflammation involve interrelated physiological processes^18^ and have been linked with increased risk of cardiovascular disease,^19–22^ type 2 diabetes^23^ and depression.^24^ HPA axis dysregulation and chronic inflammation may be triggered by exposure to psychosocial and other stressors. For example, evidence from longitudinal studies suggests that unemployment and divorce may be linked to raised chronic inflammation^1^ and altered cortisol profiles.^25^^,^^26^ Conversely, stable partnerships and work histories are likely to be beneficial for health,^27^^,^^28^ possibly acting through the preservation of optimal HPA-axis functioning and normal inflammatory responses.^29^ Although these studies do not identify a mechanism, it is possible that the health effects are at least partly mediated by differences in health behaviours and/or social position between people with distinct work and family life courses.

Sequence analysis is a novel empirical tool increasingly used in life course research to characterise progression through life course states in a given domain in a holistic manner.^30^^,^^31^ Recent availability in cohort studies of biomarkers thought to be linked with both stress and health^32^ allows for the study of entire life course biographies in relation to objective health outcomes. This study uses multi-channel sequence analysis (MCSA) to characterise work, partnership and parenthood in combination across the adult life courses (ages 16–42) of both men and women in the National Child Development Study (NCDS) 1958 British birth cohort. We investigate whether work-family types involving weak ties to paid work and family are associated with raised inflammation and altered HPA axis activity, independent of early life circumstances. We also examine the role of potential adult mediators in these relationships, as well as whether associations differ for men and women.

## Methods

### Data

The NCDS recruited 17 415 babies born in one week of 1958 (98.2% of all births that week) in Great Britain (England, Scotland and Wales).^33^ Participants were surveyed at birth and ages 7, 11, 16, 23, 33, 42, 44/45, 46 and 50. Information was collected on economic, medical, developmental and social aspects of participants’ lives. At age 44/45 (the cut-off age of this study), a sub-sample of participants (*n* = 9377, 77.9% of the target) took part in a biomedical survey measuring biomarkers including inflammatory markers and cortisol.

### Measures

#### Work-family life courses

Annual work, partnership and parenthood statuses were derived between ages 16 and 42 from start and end dates of employment, partnerships and dates of birth, death and leaving home (where appropriate) of children. Work status was defined as full-time employment, part-time employment (≤ 30 h/week), full-time homemaking or other not employed (unemployed, sick, in education or other reason). Partnership status was defined as married, cohabiting, or not living with a partner. Parental status was categorised as no children in the household or youngest child aged > 16 years, youngest child in household < 5 years or youngest child in household 5–16 years. Following imputation (see below), these three domains were cross-classified to create 26 annual work-family state variables, each with 36 possible combinations of work, partnership and parenthood.

Sequence analysis was used to derive work-family life courses by measuring the distance from each individual's work-family sequence to a set of 12 ‘ideal types’. The ‘ideal types’ were specified based upon previous knowledge of this cohort and with a view to including as much variation across genders as possible while maintaining adequate power (Table 1). Distances were calculated using the Dynamic Hamming approach^34^ and participants were categorised based upon their closest ‘ideal type’. Further information is available in Supplementary Data (see Supplementary Data online) and in McMunn *et al.*^35^

**Table 1.** Sample distributions for work-family life course types and associated ‘ideal type’ sequences

Work-family type	Men %^a^ (*N* = 3532, 48.9%)^b^	Women %^a^ (*N* = 3696, 51.1%)^b^	‘Ideal type’ sequence
‘Work, later family’	34.4	8.9	Continuous full-time employment; cohabiting mid-20s, married from late 20s; children from early 30s
‘Work, cohabitation, later parent’	6.5	5.1	Continuous full-time employment; cohabiting from mid-20s; children from early 30s
‘Work, marriage, non-parent’	7.8	8.9	Continuous full-time employment; married from early 20s; no children
‘Work, early family’	31.9	11.7	Continuous full-time employment; married and children from early 20s
‘Later family, work break’	0.2	14.0	Employed full-time until late 20s, homemaking from early 30s; married from mid-20s; children from early 30s
‘Work, no family’	12.8	10.1	Continuous full-time employment; no partner or children
‘Early family, work break’	0.1	15.8	Employed full-time until early 20s, homemaking from early-late 20s, employed part-time from early 30s; marriage and children from early 20s
‘Part-time work, early family’	0.3	18.0	Employed full-time until early 20s, part-time employed from early 20s; marriage and children from early 20s
‘No paid work, early family’	0.1	3.3	Employed part-time until early 20s, homemaking from early 20s; marriage and children from early 20s
‘Lone parent, divorced’	4.2	2.5	Continuous full-time employment; married from early 20s-late 30s, single from late 30s; children from early 20s
‘Teen parent’	0.8	1.2	Homemaker until mid-20s, employed full-time from mid-20s; married from early 30s; children from late teens
‘Unstable work, no family’	0.9	0.6	Working intermittently; no partner or children

a Percentages given as data are imputed therefore *N*s vary across imputed datasets.

b Sample restricted to those with at least one outcome.

#### Inflammation

**Figure 1.**
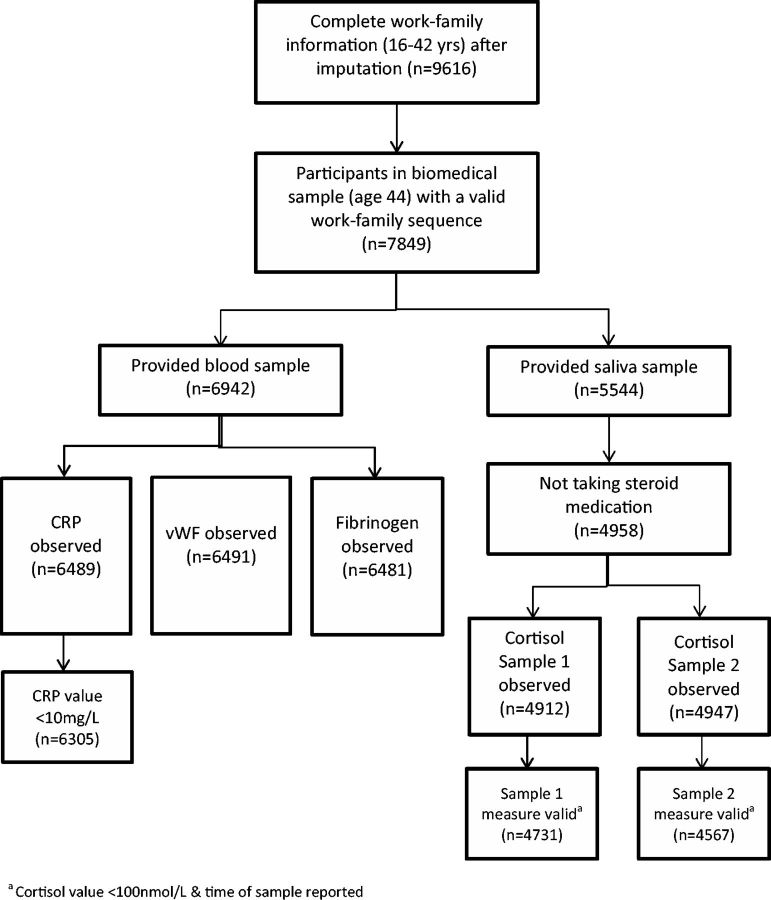
Sample selection process.

Three inflammatory markers were available from blood samples at age 44/45—C-reactive protein (CRP), von Willebrand factor (vWF) and fibrinogen. All were positively-skewed and therefore log-transformed. Participants with CRP values ≥ 10 mg/l, indicative of recent pathology or trauma,^36^ were excluded from analyses following imputation (*n* = 184, described below). Figure 1 shows how the final sample for each inflammatory and cortisol outcome was obtained.

#### Cortisol

Participants were asked to collect two saliva samples—one 45 min after waking (t1) and a second 3 h later (t2)—to capture the post-waking peak and subsequent decrease of the diurnal cortisol pattern. Time at waking (mean: 07.23 h) and times of collection (mean t1: 08.11 h, mean t2: 11.16 h) were reported. Those on steroid medication (*n* = 586) were excluded; t1 and t2 values were truncated at 100 nmol/l (*n* = 181 for t1 and *n* = 380 for t2 excluded) as values above this are implausible.^37^ Values at t1 and t2 were predicted using linear regression to take account of waking time and time since waking. Both measures were log-transformed to reduce positive skew. Cortisol was analysed in four ways: predicted values at t1 (to indicate hypo- or hypersecretion); predicted values at t2; change in cortisol between t1 and t2 (‘slope’—a flat slope is suggestive of reduced reactivity); and area-under-the-curve (AUC—greater AUC equates to greater exposure to cortisol over time), as previously described for these data.^37^

#### Covariates

Two groups of covariates were considered—early life factors (child health, socioeconomic position (SEP) and educational attainment) and adult mediators (adult SEP, health behaviours and body mass index (BMI)). The Rutter behaviour scales A (mother-reported) and B (teacher-reported) were used as indicators of emotional health at age 16. Both scales were standardised as z-scores. At age 16 the school doctor reported important health conditions present in the cohort members. These conditions included: epilepsy; physical or learning disability; musculoskeletal problems; cardiac problems; diabetes; sensory impairments; and respiratory, urogenital, gastrointestinal, haematological or CNS disorders. The likelihood that each condition, if present, might affect future employment was reported. We found that these conditions were not associated with inflammatory markers or cortisol, and were therefore not considered. Childhood SEP was indicated by father's occupational class at child's age 16 using the Registrar General's Social Class (RGSC) schema and coded as follows: professional (I); managerial and technical (II); skilled non-manual (IIINM); skilled manual (IIIM); semi-skilled manual (IV); and unskilled (V). Educational attainment was measured as the highest qualification achieved by age 23 and categorised as: no qualifications; Certificate of Secondary Education (CSE) or Ordinary-level (O-level); Advanced-level (A-level); or higher qualification or degree. For comparison, CSE/O-level qualifications are akin to US high school (or general equivalency) degree, A-level is similar to ‘some college’ and higher qualification or degree is analogous to a completed bachelor's degree or higher.^38^

Adult health behaviours and SEP have been linked to both work and family histories, as well as to inflammation and cortisol outcomes,^39–44^ and are all likely to be mediators in the present study. Adult SEP was indicated by the highest occupational class of the cohort member or their partner (where present), and was taken at age 42 (RGSC) using the same categories detailed above. Adult health behaviours at age 42 were smoking status (never, ex- or current), exercise frequency (> 3 times/week, 1–3 times/week or < 1 time/week) and harmful drinking (AUDIT questionnaire:^45^ 8+ was indicative of harmful drinking). BMI was assessed at age 42 and, despite being self-reported, was found to correlate highly with measured BMI from preceding (*r* = 0.739) and subsequent (*r* = 0.821) waves.

### Statistical analysis

#### Missing data

Missing information on work, partnership and parenthood was imputed using a recommended method to overcome problems of collinearity and inaccurate estimation of missing sequence data;^46^ 20 imputed datasets were created, imputing information for 7849 participants in the biomedical survey at age 44/45 (83.7%). Multiple imputation by chained equations was then conducted to impute covariates. The approach of imputation then deletion^47^ was used whereby all variables were imputed for all cases before excluding those with missing data on the outcomes. Further information is provided in Supplementary Data (available as Supplementary Data at *IJE* online). Descriptive analyses are presented for participants with at least one observed outcome (*n* = 7228).

#### Regression analyses

Associations between work-family groups and inflammatory and cortisol outcomes were tested in three steps using multivariate linear regression. First, the gender-adjusted association between work-family type and each outcome was estimated (Model 1). Second, early life factors were included (Model 2) to account for selection into work-family types. Third, potential adult mediators (health behaviours, BMI and SEP) were added (Model 3). The ‘Work, later family’ type was taken as the reference throughout, as strong ties to paid work and later transitions to stable family life were hypothesised to be the most ‘health enhancing’ biographical patterns. Regression estimates over the 20 imputed datasets were combined and standard errors adjusted according to Rubin's rule.^48^ R-squared terms were estimated for each model. Work-family by gender interactions were tested at each step using Wald tests, but none was statistically significant; however, we controlled for gender in all models as important differences were seen in work-family types, covariates and outcomes between men and women. Regression results are presented as percentage differences from the reference as each outcome was log-transformed.

## Results

Table 1 describes the 12 work-family types and their distribution in the NCDS. Almost all men (98%) were in one of the six work-family types characterised by continuous full-time employment. ‘Work, later family’ was the modal work-family type for men, followed closely by ‘Work, early family’; 13% of men were in the ‘Work, no family’ type, and 8% in the ‘Work, marriage, non-parent’ type. Unlike men, fewer than half (47%) of women were in one of the work-family types with continuous full-time employment. The ‘Part-time work, early family’ type was most common among women in this cohort (18%), followed by two career break groups. The two stable employment groups occupied by women were ‘Work, early family’ (12%) and ‘Work, no family’ (10%).

Table 2 shows descriptions of all analysis variables for the whole sample and stratified by work-family type. Those in the ‘No paid work, early family’ had higher levels of adolescent behavioural problems. Those with work-family life courses characterised by later transitions to parenthood (e.g. ‘Later family, work break’ and ‘Work, later family’) or no family (e.g. ‘Work, no family’) tended to have higher levels of educational attainment and more advantaged childhood SEPs. ‘Teen parents’, ‘Unstable work, no family’ and ‘Work, divorced parent’ had the highest current smoking rates, BMIs and problem drinking rates. The ‘No paid work, early family’ type was most likely to report low-intensity/no physical activity.

**Table 2.** Distribution of analysis variables by work-family type

	Whole sample	By work-family type												
		Work, later family	Work, cohabitation, later parent	Work, marriage, non-parent	Work, early family	Later family, work break	Work, no family	Early family, work break	Part-time work, early family	No paid work, early family	Lone parent, divorced	Teen parent	Unstable work, no family
*Inflammatory & cortisol outcomes*														
CRP (mg/l), median [IQR]	0.96	0.81	0.88	0.86	1.04	0.87	0.87	1.03	0.91	1.16	1.04	1.46	1.43
	[0.46, 2.19]	[0.41, 1.70]	[0.46, 1.97]	[0.44, 1.92]	[0.53, 2.16]	[0.38, 2.09]	[0.39, 1.88]	[0.43, 2.18]	[0.46, 2.29]	[0.55, 2.58]	[0.52, 1.85]	[0.61, 2.66]	[0.63, 2.65]
vWF (IU/dl), median [IQR]	117	115	118	113	120	113	116	115	113	121	123	122	135
	[92.145]	[89, 145]	[90, 146]	[90, 144]	[98, 146]	[88, 139]	[93, 146]	[93, 147]	[89, 142]	[95, 157]	[89, 150]	[99, 155]	[87, 175]
Fibrinogen (g/l), median [IQR]	2.88	2.78	2.90	2.86	2.88	2.88	2.85	3.00	2.96	3.10	2.93	3.20	2.81
	[2.52, 3.29]	[2.44, 3.16]	[2.55, 3.27]	[2.48, 3.26]	[2.53, 3.28]	[2.54, 3.30]	[2.48, 3.27]	[2.63, 3.44]	[2.62, 3.34]	[2.76, 3.51]	[2.50, 3.35]	[2.66, 3.62]	[2.51, 3.38]
t1 cortisol (nmol/l), median [IQR]	18.9	18.7	18.6	19.0	18.6	19.6	18.8	19.1	19.3	19.4	18.5	19.3	18.6
	[17.5, 20.2]	[17.5, 19.9]	[17.2, 19.9]	[17.8, 20.4]	[17.1, 20.0]	[18.4, 20.5]	[17.5, 20.1]	[17.8, 20.4]	[17.8, 20.2]	[18.2, 20.3]	[17.1, 19.9]	[17.4, 20.4]	[16.9, 19.9]
*Early life factors*														
Rutter behaviour scale, mother reported, median [IQR]	3 [2, 6]	3 [1, 5]	3 [1, 6]	3 [2, 6]	3 [2, 6]	3 [2, 5]	3 [2, 6]	3 [1, 6]	3 [2, 6]	5 [2, 7]	4 [2, 6]	4 [2, 7]	4 [2, 7]
Rutter behaviour scale, teacher reported, median [IQR]	2 [0, 5]	2 [0, 5]	3 [1, 8]	2 [0, 4]	3 [1, 6]	1 [0, 4]	2 [0, 5]	2 [0, 5]	2 [0, 5]	3 [1, 9]	3 [1, 8]	3 [1, 7]	4 [1, 9]
Father's social class, %													
I	6.2	8.1	5.6	7.6	4.1	9.7	10.0	3.0	3.7	1.7	2.7	0.9	7.9
II	22.0	26.6	20.1	21.5	17.1	27.0	27.3	18.1	19.9	16.7	19.2	18.3	19.0
IIINM	10.0	10.5	7.7	13.9	8.6	9.7	11.4	9.9	10.6	4.2	9.1	7.5	9.1
IIIM	43.6	41.5	46.9	39.7	47.8	36.9	38.3	46.2	46.5	50.1	48.9	45.5	42.7
IV	14.0	10.6	14.6	13.9	16.8	12.9	10.1	18.3	14.6	21.3	14.4	21.1	15.4
V	4.2	2.8	5.2	3.4	5.5	3.8	3.0	4.6	4.7	6.0	5.7	6.7	5.8
Educational attainment (23 years), %													
No qualifications	8.7	4.8	11.7	4.7	11.4	7.4	4.3	9.7	13.9	29.0	9.6	12.1	17.6
CSE/O-level	55.3	47.3	55.2	51.4	59.2	49.3	49.3	71.8	59.7	60.7	62.0	74.2	59.2
A-level	17.1	23.2	14.1	21.3	16.5	18.5	20.4	8.5	9.0	3.9	16.7	8.0	15.7
Higher qualification/degree	18.9	24.7	19.0	22.6	12.9	24.8	26.1	10.1	17.5	6.4	11.7	5.7	7.5
*Adult mediators*													
Household social class (42 years), %													
I	8.9	11.5	8.1	12.7	5.6	14.4	12.1	4.9	6.5	5.3	3.6	4.7	5.3
II	46.8	53.3	42.8	54.8	44.8	46.4	51.9	43.3	40.9	24.5	36.2	32.4	22.7
IIINM	21.2	18.4	20.5	19.8	22.7	15.0	19.1	26.1	28.9	15.5	23.2	19.1	23.8
IIIM	16.1	13.2	18.7	8.4	19.5	16.4	11.7	16.9	15.5	35.3	24.2	29.1	26.9
IV	5.8	3.2	8.0	3.8	5.6	6.6	4.6	7.9	6.6	17.1	10.4	11.4	13.6
V	1.2	0.4	1.9	0.5	1.7	1.3	0.6	0.8	1.7	2.4	2.4	3.4	7.7
Smoking status (42 years), %													
Never smoked	46.7	51.4	32.7	53.3	43.2	53.3	49.6	46.6	47.1	41.2	33.4	31.4	29.8
Ex-smoker	26.6	26.3	25.3	29.5	28.0	25.2	23.7	28.0	26.1	25.2	26.1	21.6	29.7
Current smoker	26.8	22.3	42.0	17.2	28.9	21.5	26.7	25.4	26.8	33.6	40.5	47.1	40.6
Harmful drinking (44 years), %													
AUDIT score < 8	74.3	70.4	67.7	74.3	68.9	89.9	70.5	86.4	87.8	87.5	61.9	63.9	66.3
AUDIT score 8+	25.7	29.6	32.3	25.7	31.1	13.1	29.5	13.6	12.2	12.5	38.1	36.1	33.7
Exercise frequency (42 years), %													
High (> 3 x per week)	25.7	21.8	23.7	25.3	24.6	32.1	25.4	28.1	28.1	31.8	30.9	34.9	32.8
Intermediate (1–3 x per week)	41.2	48.4	40.6	42.4	39.2	43.5	42.2	35.5	37.6	27.5	34.9	33.2	28.5
Low/none (< 1 x per week)	33.0	29.9	35.7	32.3	36.2	24.4	32.5	36.4	34.3	40.7	34.2	32.0	38.6
Body mass index (kg/m^2^) (42 years), mean (SD)^a^	25.7	25.8	25.3	25.4	26.6	24.6	25.1	25.4	25.4	26.0	25.7	26.3	26.9
	(4.2)	(3.7)	(3.9)	(4.3)	(4.03)	(4.1)	(4.1)	(4.7)	(4.2)	(5.2)	(4.0)	(4.9)	(5.2)

IQR, interquartile range; SD, standard deviation.

^a^Results presented for those participants with at least one outcome (*N* = 7228).

### Work-family life courses and inflammation

Associations between work-family life courses and inflammatory markers are shown in Table 3. Compared with those in the ‘Work, later family’ type, those in groups characterised by early transitions to family life (‘Work, early family’, ‘Early family, work break’, ‘Part-time work, early family’, ‘No paid work, early family’ and ‘Teen parents’) had higher CRP values (Model 1). The difference for the ‘Teen parent’ type was particularly high (88.3% higher than ‘Work, later family’). Additionally, work-family life courses characterised by partnership breakdown (‘Lone parent, divorced’) and ‘Unstable work, no family’, were also associated with raised CRP. Many of these differences were attenuated upon consideration of early life factors (Model 2); however, higher CRP levels were still seen for the ‘Work, early family’ and ‘Teen parent’ groups. After adding adult mediators (Model 3), only the ‘Teen parents’ had raised CRP at age 44/45, which was not explained by early life factors or adult mediators considered here (40.6% higher than ‘Work, later family’). Differences in CRP for the ‘Work, early family’ group were largely explained by higher average BMI in this group. The results for CRP were robust to exclusion of those taking anti-inflammatory medications (*n* = 214).

**Table 3.** Associations between work-family life courses and inflammatory markers in the NCDS, % difference (95% CIs)

	Model 1, adjusted for gender		Model 2, adjusted for gender and early life factors^a^		Model 3, adjusted for gender, early life factors and adult mediators^b^	
	% diff	95% CI	% diff	95% CI	% diff	95% CI
**CRP (*n* = 6305)**						
Work, later family	Ref		Ref		Ref	
Work, cohabitation, later parent	12.49	−1.10, 27.95	4.40	−8.18, 18.71	3.27	−8.30, 16.29
Work, marriage, non-parent	10.71	−1.17, 24.00	9.05	−2.57, 22.05	10.21	−0.75, 22.38
Work, early family	25.39	15.44, 36.19	14.90	5.76, 24.82	5.44	−2.39, 13.90
Later family, work break	1.79	−10.51, 15.79	0.06	−11.91, 13.66	1.03	−10.72, 13.77
Work, no family	3.53	−6.39, 14.51	3.63	−6.21, 14.50	5.72	−3.65, 16.00
Early family, work break	19.93	5.82, 35.92	7.72	−4.98, 22.12	4.22	−7.24, 17.08
Part-time work, early family	17.12	3.98, 31.93	6.55	−5.38, 19.98	2.65	−8.13, 14.68
No paid work, early family	40.74	11.78, 77.20	14.88	−8.68, 44.52	6.59	−13.86, 31.90
Lone parent, divorced	24.09	5.42, 46.07	12.67	−4.21, 32.52	9.72	−5.80, 27.79
Teen parent	88.28	38.43, 156.04	60.85	18.52, 118.30	40.55	5.62, 87.02
Unstable work, no family	42.29	2.60, 97.32	28.73	−7.02, 78.21	8.12	−20.24, 46.56
R-squared (%)	0.92		3.20		17.71	
F test for work-family type	5.30	*p* ≤ 0.001	2.10	*p* = 0.02	0.9	*p* = 0.54
**Fibrinogen (*n* = 6481)**						
Work, later family	Ref		Ref		Ref	
Work, cohabitation, later parent	2.73	0.35, 5.17	1.46	−0.89, 3.87	0.80	−1.40, 3.05
Work, marriage, non-parent	1.42	−0.63, 3.51	1.18	−0.85, 3.26	1.83	−0.12, 3.82
Work, early family	2.88	1.36, 4.43	1.46	−0.05, 2.99	0.38	−1.05, 1.83
Later family, work break	1.13	−1.19, 3.51	0.88	−1.43, 3.23	1.03	−1.18, 3.28
Work, no family	0.97	−0.86, 2.83	0.96	−0.85, 2.80	1.23	−0.50, 2.99
Early family, work break	4.25	1.95, 6.60	2.64	1.06, 4.97	2.15	−0.01, 4.35
Part-time work, early family	2.65	0.48, 4.87	1.13	−1.01, 3.31	0.52	−1.51, 2.59
No paid work, early family	9.82	5.40, 14.43	6.35	2.07, 10.82	4.73	0.67, 8.95
Lone parent, divorced	4.89	1.88, 7.98	3.31	0.37, 6.35	2.43	−0.36, 5.30
Teen parent	13.29	7.30, 19.62	10.40	4.63, 16.49	7.79	2.34, 13.53
Unstable work, no family	3.15	−2.68, 9.33	1.36	−4.34, 7.41	−1.28	−6.64, 4.37
R-squared (%)	2.49		4.50		14.08	
F test for work-family type	5.20	*p* ≤ 0.001	2.30	*p* = 0.01	1.70	*p* = 0.06
**vWF (*n* = 6491)**						
Work, later family	Ref		Ref		Ref	
Work, cohabitation, later parent	1.54	−2.31, 5.54	0.24	−3.57, 4.20	0.14	−3.66, 4.10
Work, marriage, non-parent	0.10	−3.27, 3.59	−0.17	−3.52, 3.31	0.17	−3.18, 3.64
Work, early family	4.53	1.97, 7.15	2.93	0.37, 5.55	2.24	−0.30, 4.84
Later family, work break	−0.12	−3.91, 3.81	−0.40	−4.17, 3.52	−0.36	−4.13, 3.55
Work, no family	1.56	−1.48, 4.69	1.55	−1.48, 4.68	1.80	−1.23, 4.92
Early family, work break	2.81	−0.96, 6.73	1.05	−2.69, 4.93	0.67	−3.06, 4.53
Part-time work, early family	−0.42	−3.92, 3.20	−1.92	−5.38, 1.66	−2.44	−5.87, 1.12
No paid work, early family	7.93	0.77, 15.60	4.34	−2.65, 11.83	3.42	−3.53, 10.87
Lone parent, divorced	3.13	−1.72, 8.22	1.45	−3.36, 6.50	1.18	−3.62, 6.22
Teen parent	8.90	−0.73, 19.46	5.79	−3.50, 15.97	4.57	−4.61, 14.63
Unstable work, no family	12.38	1.92, 23.91	10.06	−0.17, 21.35	8.32	−1.73, 19.40
R-squared (%)	0.51		1.11		2.68	
F test for work-family type	2.60	*p* = 0.003	1.50	*p* = 0.14	1.20	*p* = 0.30

diff, difference.

^a^Adjusted for gender, childhood SEP, child health, educational attainment.

^b^Adjusted for gender, childhood SEP, child health, educational attainment, smoking status, exercise frequency, problem drinking and BMI.

Similar to CRP, work-family types characterised by early parenthood, partnership breakdown and weak work ties had higher levels of fibrinogen than the ‘Work, later family’ type, as did ‘Work, cohabitation, later parent’ (Model 1). These differences were attenuated upon adjustment for early life factors (Model 2) for ‘Work, cohabitation, later parent’, ‘Part-time work, early family’ and ‘Work, early family’ groups. After including adult mediators (Model 3), fibrinogen levels remained elevated among those in the ‘No paid work, early family’ and ‘Teen parent’ types. Raised fibrinogen seen for the ‘Lone parent, divorced’ group was mediated by higher smoking rates, and lower levels of physical activity mediated raised fibrinogen previously seen for the ‘Early family, work break’ group.

With regard to vWF, those in the ‘Work, early family’, ‘No paid work, early family’ and ‘Unstable work, no family’ types had higher levels relative to the ‘Work, later family’ group (Model 1). Upon consideration of early life factors (Model 2), only those in the ‘Work, early family’ group continued to have statistically higher vWF relative to the ‘Work, later family’ group. Similar to CRP, higher vWF among the ‘Work, early family’ type was explained by higher average BMI.

### Work-family life courses and cortisol

Work-family types were associated only with t1 cortisol values (45 min after waking); therefore, only these results are presented (Table 4). Those in the ‘Later family, work break’ type had higher t1 cortisol values than those in the ‘Work, later family’ type. These differences were not explained by early life factors (Model 2), but were attenuated when adult mediators were included (Model 3). However, the fact that no other work-family groups showed associations with t1 cortisol, and no work-family groups showed associations with the other three cortisol outcomes (Supplementary Data, available as Supplementary Data at *IJE* online) points towards a null finding with stress biomarkers.

**Table 4.** Associations between work-family life courses and t1 cortisol (45 min after waking) in the NCDS, % difference (95% CIs)

	Model 1, adjusted for gender		Model 2 adjusted for gender and early life factors^a^		Model 3, adjusted for gender, early life factors and adult mediators^b^	
	% diff	95% CI	% diff	95% CI	% diff	95% CI
**t1 cortisol (*n* = 4731)**					
Work, later family	Ref		Ref		Ref	
Work, cohabitation, later parent	−0.60	−2.31, 1.13	−0.52	−2.21, 1.21	−0.23	−1.92, 1.50
Work, marriage, non-parent	0.79	−0.57, 2.17	0.86	−0.51, 2.24	0.71	−0.65, 2.10
Work, early family	−0.66	−1.70, 0.36	−0.50	−1.54, 0.55	−0.42	−1.46, 0.63
Later family, work break	1.71	0.14, 3.31	1.71	0.14, 3.31	1.40	−0.17, 2.99
Work, no family	0.01	−1.25, 1.25	0.03	−1.28, 1.22	−0.05	−1.29, 1.20
Early family, work break	−0.43	−1.95, 1.11	−0.16	−1.70, 1.40	−0.31	−1.84, 1.25
Part-time work, early family	0.002	−1.45, 1.47	0.24	−1.22, 1.72	0.21	−1.25, 1.69
No paid work, early family	0.06	−2.79, 3.00	0.41	−2.48, 3.39	0.06	−2.83, 3.03
Lone parent, divorced	−1.57	−3.79, 0.70	−1.37	−3.59, 0.91	−1.12	−3.36, 1.17
Teen parent	1.49	−2.18, 5.30	1.75	−1.93, 5.57	2.03	−1.64, 5.84
Unstable work, no family	1.07	−3.67, 6.04	1.28	−3.45, 6.25	1.47	−3.28, 6.45
R-squared (%)	3.87		4.28		5.55	
F test for work-family type	1.40	*p* = 0.18	1.20	*p* = 0.30	0.90	*p* = 0.56

^a^Adjusted for gender, childhood SEP, child health, educational attainment.

^b^Adjusted for gender, childhood SEP, child health, educational attainment, smoking status, exercise frequency, problem drinking and BMI.

## Discussion

Using data from a British birth cohort, we found that certain work-family life courses were associated with chronic inflammation in mid-life, and thus potentially greater risk of later disease.^19^ Work-family types characterised by weaker ties to paid work (‘No paid work, early family’, ‘Early family, work break’—groups comprising largely women), earlier transitions to parenthood (‘No paid work, early family’, ‘Early family, work break’, ‘Work, early family’, ‘Teen parent’), and less stable partnerships (‘Lone parent, divorced’, ‘Teen parent’) had raised inflammation in mid-life as captured by CRP, fibrinogen and vWF. This was compared with those who had children later and retained strong ties to paid work (‘Work, later family’). These associations were largely explained by less healthy lifestyles (higher rates of smoking and drinking, lower physical activity levels and higher BMI) among participants in work-family types with weaker ties to work and partnerships, and earlier transitions to parenthood. Although not working^1^^,^^49^ and partnership instability,^50^ considered separately, have been linked with chronic inflammation in previous research, ours is one of the first studies to investigate combined work-family types across the life course with objective markers of health in mid-life.

The ‘Teen parent’ group was found to have particularly high levels of CRP and fibrinogen. This is consistent with previous work from the 1946 British birth cohort which showed that an early transition to parenthood, especially one made in the teenage years, was related to higher risk of cardiovascular disease in mid-life.^51^ This association may operate through chronic inflammation,^19^ as captured in the present study. In addition, the combination of being both a teen parent and un-partnered has previously been linked to poor self-rated health,^2^^,^^52^ and our findings extend these observations to objective markers of health. Although they did not fully explain higher levels of inflammation, rates of problem drinking and current smoking were particularly high amongst ‘Teen parents’, as was average BMI. This corroborates work by Graham and colleagues^53^ who found that women who experienced single and early parenthood were more likely to be smokers than those who had children later and within a cohabiting partnership. Further work is needed to explore potential stressors linked with becoming a parent at a relatively young age, which may lead to higher levels of inflammation in mid-life that were unexplained by the measures of SEP and health behaviours included here. For example, it is possible that ‘Teen parents’ are more likely to live in polluted areas or in poor-quality housing, which we were not able to capture here. It is also possible that by the inclusion of categorical variables of problem drinking and smoking status, only part of the mediation through risky health behaviours was included.

Similarly, the mediators considered in this study did not fully explain the raised fibrinogen levels seen for the ‘No paid work, early parent’ group (a group comprising mainly women). Other studies have shown that long-term homemaking women in the 1946 British birth cohort had poorer self-rated health and obesity in mid-life, than those who had stronger ties to paid work.^3^ The current study extends this work by assessing the long-term effects of combined work and family biographies on objective markers of health in a more recent British birth cohort. We found that higher BMI and lower physical activity levels contributed most to explaining the association between ‘No paid work, early family’ and fibrinogen. However, the associations seen for ‘No paid work, early family’ and ‘Teen parents’ were not fully explained, suggesting residual confounding or mediation by other factors not considered, including environmental and physical stressors, and the accumulation of life course stress-inducing disadvantages which we have only been able to capture partially here.

Associations between work-family types and CRP were particularly large compared with those for vWF and fibrinogen. CRP is a marker of inflammatory load only, whereas vWF and fibrinogen have additional clotting and endothelial functions. In contrast to our findings on inflammation, those on t1 cortisol showed that only the ‘Later family, work break’ group had higher levels and this was found to be related to their healthier lifestyles, especially their higher levels of physical activity. However, given that no associations were seen for other work-family types and with only one of the four cortisol outcomes, the possibility of type I error cannot be ruled out. It is also possible that two measures of cortisol are insufficient to characterise diurnal patterns reliably.^54^

### Methodological considerations

In this study we were not able to take into account more detailed processes, including work and relationship quality. It has been shown that the quality of social roles may be more important for later health, than the occupation of a role.^55^ We also did not consider comorbidities in our analyses, which might influence inflammation and cortisol levels. However, data from the Health Survey for England have shown that disease prevalence is low for our age group (e.g. diabetes < 3%, ischaemic heart disease or stroke < 2%).^56^ It is also possible that type 1 errors may be present in this study, particularly regarding our four cortisol outcomes. However, this is less likely to be the case with respect to inflammatory markers across which a consistent story emerges. Also, in all likelihood mediation was not fully captured through smoking status and problem alcohol consumption, and residual confounding is likely to be present in this study. Finally, although we did not find any gender interactions, our work-family life courses are very gendered with very few men in types characterised by weak ties to paid work. Although we adjust for gender throughout, there may be some residual confounding by gender for these groups.

This study also has many strengths. Foremost is the use of multi-channel sequence analysis to model the interconnected work and family histories of both men and women—one of the first epidemiological studies to do so. The key advantage of this method is that it reduces complex and otherwise unmanageable strings of life course information—in this case, on multiple domains (work, partnership and parenthood)—to create a typology of work-family life courses that is amenable to further analysis. Moreover, the Dynamic Hamming algorithm applied in this study is particularly suited to life course epidemiology, where timing and duration are of importance. The typology we used as a guiding tool relied on the a priori specification of the ‘ideal types’. We validated this approach by comparing our groups with those created using an empirically-driven MCSA and cluster analysis. One caveat is that rare work-family histories which might have important implications for health are lost in a relatively small number of types created from all possible lived experiences. Similarly the observed associations between work-family life courses and health outcomes must be interpreted with caution, as they cannot be assumed to imply causality. That said, MCSA connects individuals’ experiences across key life domains, over time, thus providing a more holistic representation of life courses than more widely-used event-focused approaches. As such, it shows promise as a useful analytical tool for describing life course phenomena.

The longitudinal design also allowed for the consideration of previous childhood factors (such as health) which may confound associations. Despite being a birth cohort, the data are likely to be broadly representative of men and women of similar ages in Great Britain. Missing data were accounted for using multiple imputation, including an approach appropriate to categorical time series data. Finally, in contrast to many previous studies linking work and family histories to subjective or self-reported health, we used objective markers of health.

In conclusion, our study suggests that work-family life courses characterised by early entry into parenthood, combined with weak long-term ties to paid work, are associated with chronic inflammation in mid-life. Transitions to adulthood may be important, as trajectories established during this time may have long-lasting health implications.

## Funding

This work was supported by a European Research Council Starter Grant [grant number ERC-2011-StG_20101124], to A.M. (Principal Investigator). A.S. and M.K.'s time on this manuscript was partially supported by the UK Economic and Social Research Council [grant number ES/J019119/1]. P.M. and D.W. were supported by the Canadian Institutes of Health Research grant [grant number MOP 119526] and the Social Sciences and Humanities Research Council grant [grant number 43512-1267]. Open access was funded through block funding to UCL from RCUK (paid by ESRC in this instance).

**Conflict of interest:** None declared.

## Supplementary Material

Supplementary Data

Supplementary Data
