# Supplementary material for: Work-family life courses and markers of stress and inflammation in mid-life: evidence from the National Child Development Study
Source: Int J Epidemiol. 2015 Oct 14;45(4):1247–59. doi: 10.1093/ije/dyv205 (PMC5841625; doi:10.1093/ije/dyv205)
Supplement: Supplementary Data [file dyv205_supplementary_data.zip › ije-2015-01-0083-File003.docx]

**Supplement 2**

**Multiple imputation strategy**

A two-stage imputation strategy was employed in this study, both stages of which were conducted in Stata.

***Stage 1 – imputation of work-family life courses***

Missing information on work, partnership and parenthood histories was imputed using an imputation method developed by Halpin^1,2^ This method is particularly appropriate to the imputation of categorical time series data, such as sequence data, where problems with collinearity and inaccurate estimation of gaps in sequence data would be likely using more common imputation approaches, such as multiple imputation by chained equations (MICE). For instance, an individual who has been in state A for 5 years, who is then missing information for 5 years, and then are observed to be in state B for 5 years is likely to be imputed as this using the MICE approach:

AAAAA[ABABA]BBBBB

when actually the following is more likely:

AAAAA[AABBB]BBBBB

Halpin’s approach uses observed preceding and subsequent information, as these are likely to be the most reliable predictors for imputing the missing information. This imputation approach not only takes account of the states adjacent to a gap but also history – i.e. the length of time an individual has spent in these adjacent states. This approach can be applied to gaps within as well as at the beginning or end of sequences. Work, partnerships and parenthood histories were imputed separately but including the other two domains (e.g. missing work histories were imputed using partnerships and parenthood). Using Halpin’s guidance^2^ we imputed missing information for those with a maximum internal sequence gap of 13 years (50% of the total sequence length of 26 years (16-42 years)) and a maximum initial and terminal gap of 7 years (~50% of the maximum internal gap length). Following this rule, of the 9616 with complete information after imputation, 21.9% had imputed work information, 9.3% had imputed partnership information and none had imputed parenthood information^[[1]](#footnote-1)^. 20 imputed datasets were created in this first imputation stage.

Once imputed the work-family domains were combined to create 26 combined work-family variables (one for each year of interest). These work-family variables were then subjected to multi-channel sequence analysis (MCSA), which was carried out separately on each of the 20 imputed datasets and then combined.

***Stage 2 – imputation of covariates and outcomes***

The biomedical survey at age 44/45 was on a sub-sample of NCDS participants, 7849 of whom had complete work-family information between 16 and 42 years from stage 1 of the imputation process (see fig. 1). MICE was employed to impute outcomes and covariates in a second stage of imputation. The imputation model contained the derived work-family typology variable from the MCSA, all analysis variables, plus the same variables (where available) from preceding or subsequent waves e.g. father’s social class at age 11 in addition to father’s social class at age 16 used in the analyses. The multiple imputation then deletion method was employed whereby we imputed the outcome variables but only used information on individuals for whom each outcome was observed.^3^ Figure 1 provides more information on the numbers of participants with each observed outcome.

**References**

1. Halpin B. Multiple imputation for life-course sequence data [Internet]. Limerick: University of Limerick; 2012. Available from: http://www.academia.edu/2601453/Multiple_Imputation_for_Life-Course_Sequence_Data

2. Halpin B. Imputing sequence data: extensions to initial and terminal gaps, Stata’s mi [Internet]. Limerick: University of Limerick; 2013. Available from: http://www3.ul.ie/sociology/pubs/wp2013-01.pdf

3. Hippel P Von. Regression with missing Ys: An improved strategy for analyzing multiply imputed data. *Sociol Methodol*. 2007;**37**:83–117.

1. None of the NCDS participants had imputed parenthood information as those with missing information were missing too much information under the rules we set for the maximum amount that would be imputed (see above for more detail) [↑](#footnote-ref-1)
