# Supplementary material for: Work-family life courses and markers of stress and inflammation in mid-life: evidence from the National Child Development Study
Source: Int J Epidemiol. 2015 Oct 14;45(4):1247–59. doi: 10.1093/ije/dyv205 (PMC5841625; doi:10.1093/ije/dyv205)
Supplement: Supplementary Data [file dyv205_supplementary_data.zip › ije-2015-01-0083-File004.docx]

**Supplement 3 – Associations between work-family life courses and other cortisol outcomes (t2, slope and AUC measures) in the NCDS, % difference (95% CIs)**

|  | **Model 1 - adj. gender** | | **Model 2 - adj. gender & early life factors^1^** | | **Model 3 – adj. gender, early life factors & adult mediators^2^** | |
| --- | --- | --- | --- | --- | --- | --- |
|  | **% diff** | **95% CI** | **% diff** | **95% CI** | **% diff** | **95% CI** |
| **t2 cortisol (n=4567)** |  |  |  |  |  |  |
| Work, later family | Ref |  | Ref |  | Ref |  |
| Work, cohabitation, later parent | -1.00 | -2.38, 0.40 | -0.94 | -2.32, 0.46 | -0.72 | -2.10, 0.68 |
| Work, marriage, non-parent | 0.27 | -0.88, 1.43 | 0.31 | -0.84, 1.48 | 0.19 | -0.96, 1.36 |
| Work, early family | -0.80 | -1.67, 0.07 | -0.71 | -1.59, 0.18 | -0.63 | -1.52, 0.24 |
| Later family, work break | 1.02 | -0.31, 2.36 | 1.03 | -0.30, 2.37 | 0.74 | -0.58, 2.08 |
| Work, no family | -0.50 | -1.54, 0.56 | -0.51 | -1.56, 0.55 | -0.53 | -1.57, 0.53 |
| Early family, work break | -0.68 | -1.96, 0.62 | -0.54 | -1.84, 0.77 | -0.32 | -1.95, 0.65 |
| Part-time work, early family | -0.36 | -1.58, 0.88 | -0.21 | -1.44, 1.04 | -0.32 | -1.46, 1.03 |
| No paid work, early family | -0.50 | -2.88, 1.94 | -0.26 | -2.66, 2.22 | -0.58 | -2.99, 1.89 |
| Lone parent, divorced | -1.33 | -3.03, 0.60 | -1.08 | -2.88, 0.75 | -0.93 | -2.73, 0.91 |
| Teen parent | 0.81 | -2.30, 4.02 | 0.95 | -2.17, 4.17 | 1.18 | -1.94, 4.38 |
| Unstable work, no family | 0.86 | -2.62, 4.57 | 1.04 | -2.55, 4.77 | 1.17 | -2.45, 4.90 |
| R-squared (%) | 6.53 |  | 6.81 |  | 7.98 |  |
| F test for work-family type | 1.34 | p=0.20 | 1.18 | p=0.29 | 0.90 | p=0.54 |
| **Slope (n=4395)** |  |  |  |  |  |  |
| Work, later family | Ref |  | Ref |  | Ref |  |
| Work, cohabitation, later parent | -2.98 | -15.65, 1.10 | -2.14 | -13.75, 11.03 | -3.10 | -14.58, 9.94 |
| Work, marriage, non-parent | -2.44 | -10.33, 6.14 | -2.19 | -10.11, 6.44 | -1.50 | -9.49, 7.20 |
| Work, early family | 2.46 | -3.91, 9.24 | 3.47 | -3.03, 10.41 | 3.24 | -3.26, 10.18 |
| Later family, work break | -6.45 | -15.06, 3.03 | -6.50 | -15.11, 2.97 | -4.93 | -13.70, 4.74 |
| Work, no family | 2.41 | -5.23, 10.68 | 2.67 | -4.99, 10.95 | 2.89 | -4.77, 11.19 |
| Early family, work break | -0.84 | -8.65, 9.05 | -0.05 | -9.20, 10.02 | 0.74 | -8.49, 10.90 |
| Part-time work, early family | -0.01 | -8.66, 9.46 | 0.47 | -8.27, 10.05 | 0.70 | -8.07, 10.31 |
| No paid work, early family | -2.36 | -18.04, 16.54 | -0.11 | -16.31, 19.26 | 2.29 | -14.37, 22.20 |
| Lone parent, divorced | 2.25 | -15.00, 23.01 | 3.40 | -14.00, 24.33 | 2.98 | -15.57, 24.15 |
| Teen parent | -2.47 | -22.22, 22.30 | -0.25 | -20.48, 25.14 | -1.18 | -21.19, 23.91 |
| Unstable work, no family | -14.44 | -44.24, 31.30 | -12.83 | -42.99, 33.29 | -13.31 | -43.49, 32.98 |
| R-squared (%) | 6.84 |  | 7.27 |  | 7.88 |  |
| F test for work-family type | 0.46 | p=0.93 | 0.51 | p=0.90 | 0.43 | p=0.94 |
| **AUC (n=4395)** |  |  |  |  |  |  |
| Work, later family | Ref |  | Ref |  | Ref |  |
| Work, cohabitation, later parent | -0.92 | -2.46, 0.65 | -0.79 | -2.33, 0.77 | -0.47 | -2.00, 1.09 |
| Work, marriage, non-parent | 0.60 | -0.64, 1.86 | 0.67 | -0.58, 1.94 | 0.54 | -0.70, 1.80 |
| Work, early family | -0.73 | -1.66, 0.22 | -0.55 | -1.50, 0.41 | -0.43 | -1.39, 0.53 |
| Later family, work break | 1.48 | -0.01, 2.91 | 1.49 | 0.07, 2.92 | 1.20 | -0.21, 2.63 |
| Work, no family | -0.11 | -0.01, 1.03 | -0.10 | -1.23, 1.03 | -0.04 | -1.16, 1.10 |
| Early family, work break | -0.74 | -1.23, 0.65 | -0.49 | -1.87, 0.91 | -0.57 | -1.96, 0.81 |
| Part-time work, early family | -0.24 | -2.10, 1.10 | -0.01 | -1.33, 1.34 | 0.02 | -1.31, 1.36 |
| No paid work, early family | 0.27 | -1.55, 2.92 | 0.71 | -1.91, 3.40 | 0.38 | -2.22, 3.05 |
| Lone parent, divorced | -1.18 | -2.30, 0.91 | -0.93 | -2.99, 1.16 | -0.72 | -2.79, 1.40 |
| Teen parent | 1.67 | -1.64, 5.09 | 1.88 | -1.44, 5.31 | 2.09 | -1.21, 5.51 |
| Unstable work, no family | -1.13 | -4.86, 2.75 | -0.81 | -4.55, 3.06 | -0.61 | -4.37, 3.30 |
| R-squared (%) | 1.04 |  | 1.48 |  | 2.73 |  |
| F test for work-family type | 1.59 | p=0.10 | 1.31 | p=0.21 | 0.93 | p=0.51 |

^1^Adjusted for gender, childhood SEP, child health, educational attainment;

^2^Adjusted for gender, childhood SEP, child health, educational attainment, smoking status, exercise frequency, problem drinking, and BMI
