# Supplementary material for: Work-family life courses and markers of stress and inflammation in mid-life: evidence from the National Child Development Study
Source: Int J Epidemiol. 2015 Oct 14;45(4):1247–59. doi: 10.1093/ije/dyv205 (PMC5841625; doi:10.1093/ije/dyv205)
Supplement: Supplementary Data [file dyv205_supplementary_data.zip › ije-2015-01-0083-File002.docx]

**Supplement 1**

**Sequence analysis**

Sequence analysis, and in particularly multi-channel sequence analysis (MCSA), is a novel method in life course social epidemiology. The twelve ‘ideal types’ detailed in table 1 were devised independently by the first and last author and then compared and found to overlap substantially. These ‘ideal types’ were based upon prior knowledge of men’s and women’s work and family arrangements in this cohort and wider societal trends. The ‘ideal types’ were added to the dataset as ‘invented’ cases and the distance between every participant’s work-family life course was measured from that of each ‘ideal type’. Different distance measures can be used in this stage of the sequence analysis. We decided to use Lesnard’s^1^ Dynamic Hamming algorithm which is particularly appropriate to the current study and a life course approach. This method takes into account the timing of work, partnership and parenthood transitions. In particular it assigns higher costs to substitutions that parallel transitions made at times when relatively few participants are changing states. Conversely, lower costs are assigned to substitutions paralleling transitions made at times when many participants are changing states (e.g. in late adolescence). Dynamic Hamming sequence analysis was implemented using the –seqcomp- plug-in in Stata^2^.

Participants were then allocated to the ‘ideal type’ group which was closest to their actual work-family life course, thereby creating a single work-family type variable with twelve categories. This was then used in subsequent analyses reported in this study. The validity of the ‘ideal type’ classification was assessed by examining within- and between-group variability. Work-family types were added or removed at this stage based on these validity measures. For instance, a ‘Step-Dads’ type was removed as very few participants were allocated to this work-family type and participants who were allocated were found to almost equally as close to another work-family type.

In addition, an empirically-driven sequence analysis method, involving cluster analysis by Ward’s linkage, was used to validate our theoretically derived work-family types. This empirical method compares each participant’s work and family life course to each other participant’s (rather than to a series of ‘ideal types’). A 12 cluster solution produced a work-family typology which was very similar to that produced using our method.

Sequence analysis therefore produces a single variable of a work-family typology which can be used in subsequent analyses, such as those presented in this study.

**References**

1. Lesnard L. Optimal matching and social sciences. 2006; Available from: http://laurent.lesnard.free.fr/article.php3?id_article=14

2. Lesnard L. seqcomp, a sequence analysis Stata plug-in [Internet]. 2008. Available from: http://laurent.lesnard.free.fr/article.php3?id_article=8
