# Supplementary material for: Work-family life courses and markers of stress and inflammation in mid-life: evidence from the National Child Development Study
Source: Int J Epidemiol. 2015 Oct 14;45(4):1247–59. doi: 10.1093/ije/dyv205 (PMC5841625; doi:10.1093/ije/dyv205)
Supplement: Supplementary Data [file supp_45_4_1247__index.html]

Work-family life courses and markers of stress and inflammation in mid-life: evidence from the National Child Development Study — Work-family life courses and markers of stress and inflammation in mid-life: evidence from the National Child Development Study — Supplementary Data 

# Work-family life courses and markers of stress and inflammation in mid-life: evidence from the National Child Development Study

## Supplementary Data

files

- Supplementary Data - docx file
- Supplementary Data - docx file
- Supplementary Data - docx file
